# Demography of avian scavengers after Pleistocene megafaunal extinction

**DOI:** 10.1038/s41598-019-45769-w

**Published:** 2019-07-04

**Authors:** Paula L. Perrig, Emily D. Fountain, Sergio A. Lambertucci, Jonathan N. Pauli

**Affiliations:** 1 Department of Forest and Wildlife Ecology, University of Wisconsin-Madison, 1630 Linden Dr., Madison, Wisconsin 53706 USA; 2 Grupo de Investigaciones en Biología de la Conservación, Laboratorio Ecotono, INIBIOMA (CONICET-Universidad Nacional del Comahue), Quintral 1250, Bariloche, Rio Negro 8400 Argentina

**Keywords:** Community ecology, Conservation biology, Palaeoecology

## Abstract

The late Quaternary megafauna extinctions reshaped species assemblages, yet we know little about how extant obligate scavengers responded to this abrupt ecological change. To explore whether obligate scavengers persisted by depending on contemporary community linkages or via foraging flexibility, we tested the importance of the trophic interaction between pumas (*Puma concolor*) and native camelids (*Vicugna vicugna* and *Lama guanicoe*) for the persistence of Andean condors (*Vultur gryphus*) in southern South America, and compared the demographic history of three vultures in different continents. We sequenced and compiled mtDNA to reconstruct past population dynamics. Our results suggest that Andean condors increased in population size >10 KYA, whereas vicuñas and pumas showed stable populations and guanacos a recent (<10 KYA) demographic expansion, suggesting independent trajectories between species. Further, vultures showed positive demographic trends: white-backed vultures (*Gyps africanus*) increased in population size, matching attenuated community changes in Africa, and California condors (*Gymnogyps californianus*) exhibited a steep demographic expansion ~20 KYA largely concurrent with North American megafaunal extinctions. Our results suggest that dietary plasticity of extant vulture lineages allowed them to thrive despite historical environmental changes. This dietary flexibility, however, is now detrimental as it enhances risk to toxicological compounds harbored by modern carrion resources.

## Introduction

Historical species assemblages can provide insight into the contemporary structure and functioning of communities^1^. Pleistocene communities, in particular, sustained diverse vertebrate assemblages of mega-carnivores and -herbivores (average body size ≥44 kg)^2,3^ that provided plentiful carrion resources for scavenging^4^. These carrion resources precipitated a rapid radiation of avian obligate scavengers^5–7^ that varied in morphology and body size according to different feeding strategies^8^. Climatic changes and human impacts during the Pleistocene-Holocene transition triggered a massive loss of megafauna^9–11^: only ~5% of megaherbivores, 40% of megacarnivores^12^, and ~41% of obligate scavengers^7,13^ genera persisted. The near-complete disassembly of Pleistocene communities radically transformed ecological interactions for those species that went through the extinction epoch^1^. Yet, the mechanisms by which species survived ecological changes are poorly understood, especially for guilds tightly linked to mega-mammals.

The Pleistocene megafaunal extinctions were a globally heterogeneous phenomenon^12,14^. Across Africa and southeast Eurasia, where large-bodied species had a deep history of coevolution with hominids, loss of megafauna was relatively moderate and gradual^15^. For example, in Sub-Saharan Africa most of the Pleistocene megafauna persist today, including 31 genera of mega-herbivores and 5 mega-carnivores^12^. Unsurprisingly, then, Africa (*n* = 11) and southern Asia (*n* = 10) support the greatest diversity of the largest obligate scavengers^16^ which rely upon carrion from herds of ungulates that die primarily from non-predatory causes^17,18^. In contrast, the megafauna of the Americas experienced a punctuated wave of extinctions, losing 70–80% of Pleistocene megafauna genera^12,19^ from a combination of climate changes and human arrival^9,10,20^. In North America, this extinction wave drove the disappearance of at least seven genera of vultures^21,22^. The California condor (*Gymnogyps californianus*), however, persisted into the Holocene by relying on marine-derived food resources^23,24^.

The megafauna extinctions in South America were more extensive than on any other continent with the complete loss of mega-herbivores, 50% of megacarnivores^12,25^, and at least 50% of vultures^26–28^. Notably, however, a tightly-linked community module of pumas (*Puma concolor*) preying largely on wild camelids, guanacos (*Lama guanicoe*) and vicuñas (*Vicugna vicugna*), emerged in Patagonia and the southern Andes^29^. Moreover, these species exhibited a demographic expansion (increased effective population size) in the mid-Holocene^30–32^, leading to a rapid reorganization of the ecological community to one that became dominated by mid-sized vertebrates^29,33^. The largest extant scavenger of South America, the Andean condor (*Vultur gryphus*), currently relies almost exclusively on terrestrial food resources^34,35^. In areas where native ungulates have been extirpated, Andean condors now forage on an array of exotic prey (livestock [*Ovis aries*, *Bos taurus*], European hares [*Lepus europaeus*], red deer [*Cervus elaphus*])^36,37^. Comparatively, in pristine landscapes Andean condors show a strong dependency on puma-killed camelids, indicating a tight ecological association among these species^34^. It is unknown, however, whether this trophic linkage to the puma-camelid predatory interaction occurred historically and played a role in sustaining Andean condors during the Pleistocene extinctions. Further, it is unknown how these two different contemporary foraging strategies affect the long-term viability and persistence of Andean condor populations.

The demography of obligate scavengers are generally linked to the availability of carrion resources^38^. Apex predators can impact the abundance of scavenger populations by hunting large prey continuously through time, thus providing spatially and temporarily reliable access to carrion^39–41^. If necrophagous birds rely on predators provisioning the carrion of mammalian herbivores, their historic demographic trajectories should mirror that of the species they depended upon. Alternatively, if carrion availability is from other resources (e.g., marine^24^) or other sources of ungulate mortality (e.g., malnutrition, disease, extreme weather events^18^), there should be independent population size changes between predators, scavengers and ungulates. To test these competing hypotheses, we first explored past trophic linkages in South American communities. We hypothesized that if the puma-camelid predator-prey interaction sustained Andean condor populations in southern South America from the early Holocene to historical times, the demographic trajectories for Andean condors, pumas, vicuñas and guanacos would be coupled and have expanded synchronously in the early Holocene. On the other hand, if Andean condors persisted through the Pleistocene extinctions by consuming other dietary resources, their paleo-demographic trajectories would not be synchronized with those of pumas, vicuñas and guanacos, indicating that Andean condors persisted into the Holocene thanks to a plastic foraging behavior. Secondly, we reconstructed the demographic trajectory of other vulture species that share similar life history strategies to Andean condors, but experienced different historical changes to their communities: white-backed vultures (*Gyps africanus*) in Africa and California condors in North America. Given that white-backed vultures are dependent on African ungulates, which experienced relatively attenuated community changes in the late Pleistocene^15,18^, we predicted that these vultures will exhibit a relatively stable population size over time. For California condors, which persisted through the late Quaternary extinctions by shifting from terrestrial to marine food resources^24^, we predicted that they would exhibit similar historical demographic dynamics to Andean condors given that both species had to rely upon alternative food resources after the collapse of megafauna. To test our predictions, we implemented three complementary analytical methods that infer past population dynamics from contemporary gene sequences. In particular, we sequenced mitochondrial (mtDNA) and nuclear loci (nDNA) of Andean condors and compiled available mtDNA sequences from GenBank to study changes in population size of pumas, Andean condors, vicuñas, guanacos, California condors and white-backed vultures via neutrality tests, mismatch distributions, and Extended Bayesian Skyline Plot coalescent models (EBSP).

## Results

**Figure 1:**
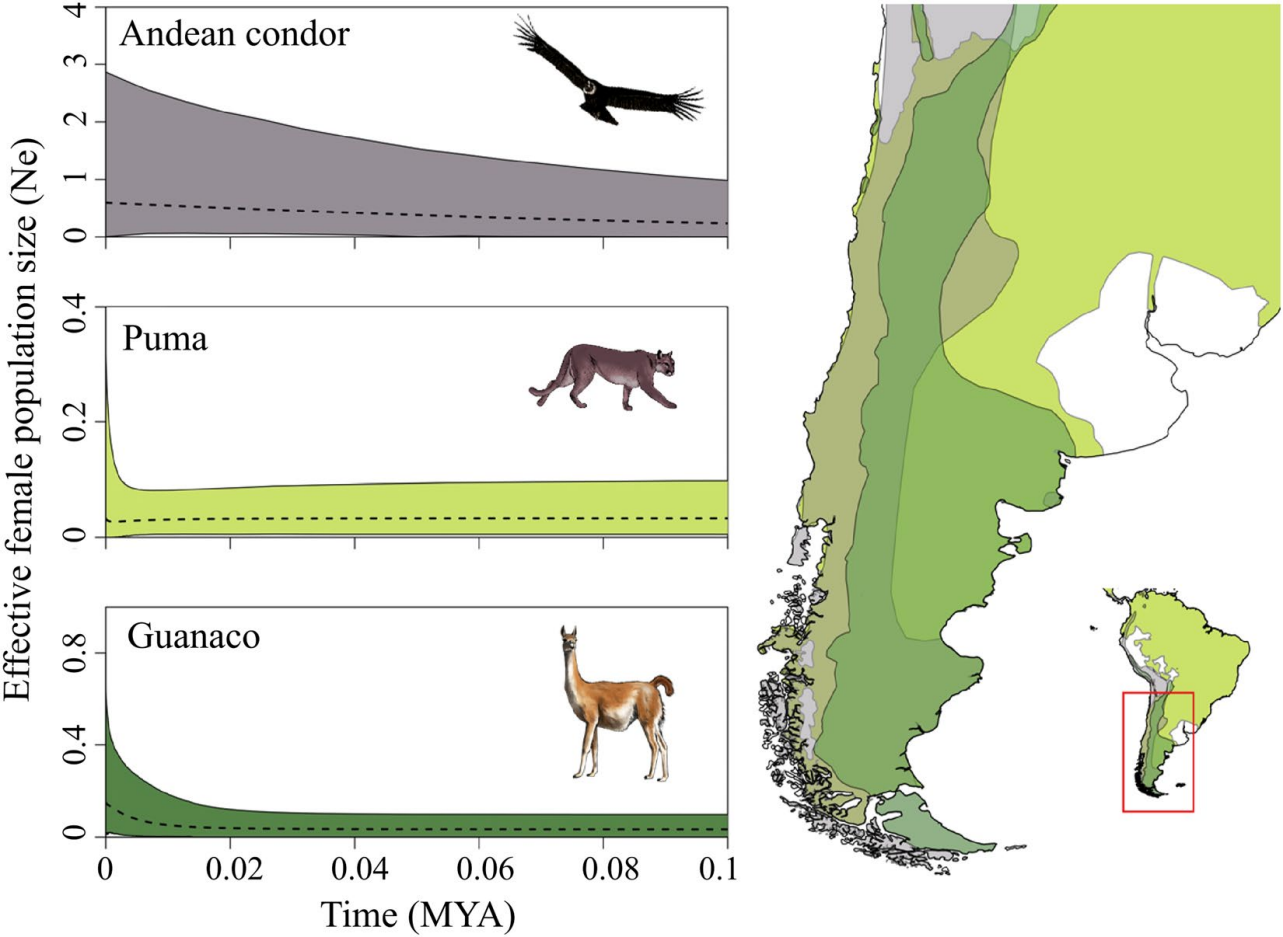
Extended Bayesian Skyline Plots illustrating female effective population size by generation time on a scale of millions of years (MYA) using combined nuclear and mitochondrial DNA sequences of Andean condors, and mitochondrial DNA sequences of pumas and guanacos in southern South America. Dotted line depicts median values; shaded region represents 95% highest posterior density.

For Andean condors, we amplified 522–538 base pairs of the c-myc gene, and 1502–1639 bp of mitochondrial control region and partial 12S (Table 1). The c-myc nuclear gene presented only one variable nucleotide, resulting in low haplotype diversity (0.09) and insignificant values of neutrality test statistics (Table 2). CR1 showed the highest number of variable nucleotides compared to CR2 and 12S (21 vs 12 and 4) and the highest haplotype diversity (0.88 vs 0.087 and 0.17, Supplementary Table S1). Neutrality tests with concatenated mitochondrial and nuclear loci were significant and negative (Table 1), indicating population expansion of Andean condors. A unimodal distribution of pairwise differences from mismatch analysis (Supplementary Fig. S1) also indicated that a demographic expansion affected neutrality, with an estimated time since expansion of ~12 KYA (τ = 0.732). The EBSP plot exhibited a slight and steady increase in population size since ~100 KYA (Fig. 1), and the majority of EBSP chains detected one or more population changes (Supplementary Fig. S2). Yet, the null hypothesis of population stability could not be rejected since the 95% Highest Posterior Density (HPD) of population changes included zero (95% HPD 0–3, Supplementary Fig. S2).

**Table 1:** Number of samples (n), gene length in base pairs (bp), reference for samples obtained via GenBank, estimated median and 95% highest probability density (HPD) mutation rate for each species, and model parameters for Extended Bayesian Skyline Plot analyses (evolutionary model for the sequence, mean in real space and standard deviation for log normal clock prior, and generation length and sampling interval of Markov Chain Monte Carlo chains).

Species	*n*	Gene	GenBank	Bp	Mutation rate	EBSP model parameters		
						Evol. model	Clock prior; mean (sd)	MCMC
Andean condor	23	CR1		621–622	0.0129 (0.0006–0.0295)	HKY + I^a^	0.013 (0.6)	10 × 10^8^; 25 × 10^2^
		CR2		353–479	0.0169 (0.0063–0.029)	TrN	0.025 (0.45)	
		12S		528–538	0.0031 (0.00051–0.0063)	F81	0.003 (0.6)	
		c-myc		522–538	0.0002 (0.00003–0.0005)	F81	0.0002 (0.7)	
California condor	75	D-loop	D’Elia *et al*.^58^	569	0.0129 (0.0006–0.0296)	HKY + I^b^	0.013 (0.5)	10 × 10^8^; 3 × 10^3^
White-backed vulture	77	Cyt-b	Arshad *et al*.^61^	1026	0.014 (0.0003–0.046)	HKY	0.014 (1.2)	9 × 10^7^; 25 × 10^2^
Puma	45	NAD5	Matte *et at*.^30^	313	0.0221 (0.0080–0.0464)	TN93	0.022 (0.4)	9 × 10^7^; 25 × 10^2^
	22	ATP8	Culver *et al*.^47^	191	0.0155 (0.006–0.0322)	HKY	0.015 (0.5)	
Vicuña	23	CR	Marin *et al*.^32^	513	0.0379 (0.0112–0.0929)	HKY + I^c^	0.04 (0.63)	8 × 10^7^; 25 × 10^2^
Guanaco	196	D-loop	Marin *et al*.^31^	442	0.0493 (0.0238–0.0841)	JC	0.05 (0.3)	26 × 10^7^; 10 × 10^2^

^a^Proportion invariant = 0.868.

^b^Proportion invariant = 0.805.

^c^Proportion invariant = 0.929.

**Table 2:** Results of Tajima’s *D* (D_T_), Fu’s *Fs* and Fu and Li’s D* (D_FL_) neutrality tests under assumptions of constant population size, and *p* - values from 10,000 coalescent simulations. Statistically significant results are shown in bold.

Species	D_T_	p-value	Fu’s Fs	p-value	D_FL_	p-value
Andean condor - complete	−1.68	**0**.**01**	−3.6	**<0**.**01**	−2.21	**0**.**02**
Andean condor - mtDNA	−2.06	**<0**.**01**	−7.50	**<0**.**01**	−2.40	**0**.**02**
Andean condor - nDNA	−1.16	0.27	−0.99	0.35	−1.59	0.06
California condor	−1.49	**0**.**05**	−9.44	**<0**.**01**	−4.76	**<0**.**01**
White-backed vulture	−1.59	**0**.**03**	−7.33	**<0**.**01**	−1.49	0.09
Puma	1.98	0.97	2.43	0.88	1.01	0.86
Vicuña	1.79	0.98	−0.29	0.47	1.16	0.95
Guanaco	−2.33	**<0**.**01**	−21.84	**<0**.**01**	−2.67	**0**.**01**

For guanacos, significant neutrality tests (Table 2), largely unimodal mismatch distributions, and EBSP analysis with 95% HPD of population changes excluding zero (Fig. 1, 95% HPD 1–4) corroborated previous reports of guanacos undergoing a recent population expansion (<10 KYA). However, we did not find this pattern for vicuñas and pumas inhabiting southern South America. In particular, pumas yielded positive neutrality statistics (Table 2), a bimodal mismatch distribution (Supplementary Fig. S1) and a flat EBSP plot with 95% HPD including zero (Supplementary Fig. S2), overall indicative of a stable population size. Similar results were obtained for vicuñas in their current southern range (Table 2; Supplementary Figs S2 and S3). The independence of the demographic histories of Andean condors, pumas, and camelids is evident even when EBSP analyses were conducted fixing molecular evolution rates at the lower, median and upper values estimated (Supplementary Fig. S4).

**Figure 2:**
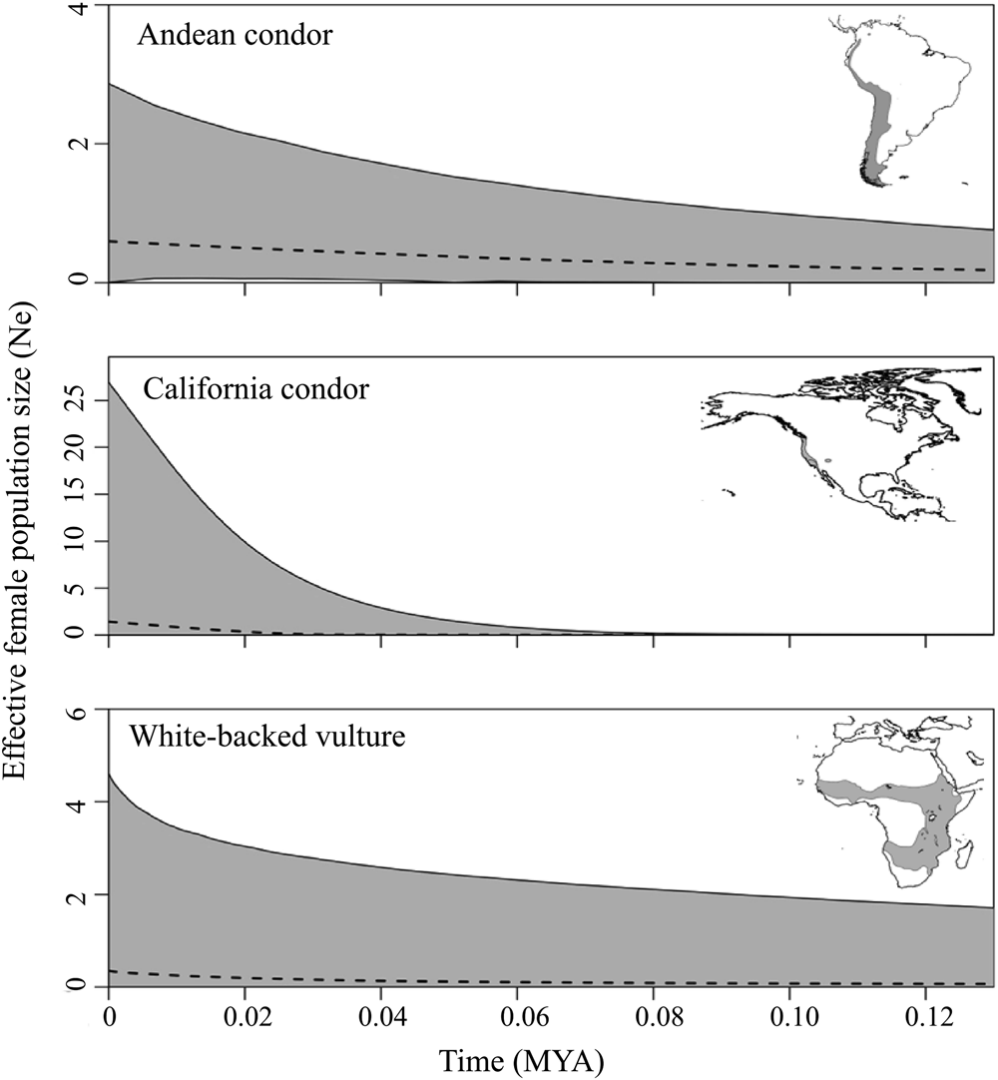
Extended Bayesian Skyline Plots illustrating female effective population size by generation time on a scale of millions of years (MYA) for Andean condors, California condors and white-backed vultures. Dotted lines depict median values; shaded region represents 95% highest posterior density.

Skyline analysis revealed population expansion of California condors since ~20 KYA (as indicated by the median effective population size [Fig. 2], and 95% HPD of population changes excluding zero) which was supported by significant neutrality tests (Table 2) and a unimodal mismatch distribution (Supplementary Fig. S1). Similarly, a smooth and unimodal mismatch analysis (Supplementary Fig. S1) along with negative and significant Fu’s and Tajima’s D values indicate that white-backed vultures experienced population expansion, although Fu and Li D* value was non-significant (Table 2). Visual inspection of the EBSP plot indicates a slightly increasing population trend since ~30 KYA (Fig. 2), although 95% HPD interval of population changes overlapped zero (Supplementary Fig. S2). Mismatch distributions suggested an expansion ~47.4 KYA (τ = 1.36).

## Discussion

We found independent historical demographic trajectories among pumas, condors and wild camelids in southern South America, and no support for the condor-puma-camelid being a historical relationship that allowed the persistence of Andean condors despite the loss of megafauna. In particular, our neutrality tests and mismatch analysis indicated an increase in Andean Condors ~12 KYA. While the 95% highest posterior density of population changes for our EBSP analysis overlapped zero^42^, we attribute this to a lack of power to detect slight demographic changes from a small sample size (n = 23)^43^. In contrast to EBSP, neutrality tests are robust to small sample sizes and reflect either the effects of natural selection or recent demographic changes^43^. Given the long generation time, low genetic diversity, and lack of population structure in the Andean condor^44,45^, we are confident that our results are driven by a demographic change and not by natural selection. In contrast to Andean condors, vicuñas and pumas showed stable populations, and guanacos a steep and recent (<10 KYA) demographic expansion. These results corroborate previous work that have shown increasing guanaco populations during the early-Holocene^31^ and nominal change for vicuñas when restricted to southern South America^32^. It is notable that vicuña populations in northern Peru exhibited recent demographic expansion (Supplementary Fig. S2)^32,46^. Our finding that pumas were stable during the late Pleistocene-Holocene differs from previous work showing demographic expansion of South American pumas^30^. We attribute these different conclusions to sampling design: while Matte *et al*.^30^ combined puma samples from across the continent – pooling five subspecies^30,47^ – we targeted pumas within Argentina and Chile. It is possible that relatively moderate changes in total mammal biomass^48,49^ along with expanding human populations that competed with pumas for prey^50,51^, prevented significant demographic changes in the puma population. Accurate inference of the timing of historical demographic change relies on estimating a species-specific substitution rate^43^. As a consequence of the previously reported time-dependency of mitochondrial substitution rates^52^, the timing of events inferred in our study (including the ~12 KYA onset of expansion in Andean condors) may be overestimated. Regardless, our discordant neutrality tests (Table 2), mismatch analyses (Supplementary Fig. S1) and Skyline-plots (Fig. 2), all point to independent demographic histories of Andean condors, pumas and camelids. Thus, carrion subsidies from puma-killed camelids do not seem to explain the demographic trajectory of Andean condors, suggesting that food availability was not a limiting factor for its population in southern South America.

While extant lineages of Andean condors appear to have expanded along the Andes, condor populations in their eastern range went extinct after the Pleistocene^53^, and the reasons for this range contraction remain unclear. On one hand, Andean condors depend upon uplift wind to soar in search for carcasses in open habitats^54^. Thus, climatic changes could have had a great impact on condor populations^55^. In the early Holocene, hotter and more humid conditions in eastern South America resulted in decreased thermal uplifts, landscapes with more vegetative cover^55^, and faster decomposition of carrion^56^, all of which could have contributed to the loss of large soaring birds adapted to scavenging in arid environments^55^. On the other hand, Andean condor populations could have been driven to extinction by a reduced availability of carrion resources after the loss of megafauna^28^, which was the case for most North American vultures and raptors^23^. California condors were able to survive Quaternary extinctions due to marine subsidies; by the early Holocene, those condors were confined to the Pacific coast of North America where marine mammals offered the only remaining source of large animal carcasses^23,24^. It is unknown if Andean condor foraging history mirrors that of California condors. Historical samples indicate that marine remains were more important to Andean condors in the 19^th^ century compared to now^35^. Quantifying Andean condor diet during the early Holocene^24^ would help to reveal if marine subsidies were consumed and contributed to the persistence of Andean condors during the late Quaternary extinctions. Regardless of exact foraging mechanism, persisting Andean condors do not appear to have experienced a population bottleneck^44^, which is supported by archeological records indicating that the species was common across their current range until the 19^th^ century. Overall, then, reductions in extant Andean condor populations seem to be recent (<500 years), and largely caused by anthropogenic impacts^53^.

As with Andean condors in South America, our results suggest an increase in vulture populations in North America and Africa despite significant climatic and ecological changes^57^. Notably, California condors appear to have undergone a steep demographic expansion ~20 KYA, which could explain their high mitochondrial DNA diversity^58^. California condors possibly benefited from relaxed competition due to the extinction of other avian scavengers during the particularly abrupt megafaunal extinctions that occurred in North America during the Pleistocene-Holocene transition^13,59,60^. Contrary to our expectation, our neutrality tests and mismatch analysis suggested that the most widespread and common African vulture, the white-backed vulture^16^, also experienced a demographic expansion ~47 KYA, which is supported by previous studies showing high genetic diversity in historically large populations^61,62^. As with Andean condors, though, EBSP 95% highest posterior density of population changes overlapped zero (Supplementary Fig. S2), which we attribute to low diversity in the gene fragment analyzed. Losses of African megafauna were substantial but happened earlier than the period studied^12^, so white-backed vultures possibly benefited from a constant carrion supply from wild ungulates^18^. Our analyses, then, suggest that extant vultures – in the Americas and Africa – not only persisted but increased in population size despite large ecological shifts.

Most vertebrates that survived the last Quaternary extinction possessed flexible foraging behaviors^59^. Indeed, extinct vultures were generally larger and possessed more extreme skull morphologies compared to extant species, indicating that intermediate sized scavengers were more likely to survive into the Holocene^8^. Extant vultures show high flexibility in foraging, as evidenced by their ability to exploit small carcasses^36,37,63,64^ and a diversity of human-related carrion resources^65,66^. As human land-use intensifies, vultures have increasingly taken advantage of novel food sources. Unfortunately, these new foraging opportunities are often associated with toxicological risks, such as lead from hunted animals^67^, pharmaceutical compounds in livestock^68^, or poison intentionally deployed on carrion remains^69^. Further, vultures’ consumption of human-related food resources results in direct persecution. Both dietary toxins and persecution associated with current-day carrion sources are the main threat for vultures worldwide^70^. Thus, the flexible foraging strategy appears to be a “double-edged sword” – a behavioral trait that enabled lineages of vultures to persist through the Pleistocene epoch but now enhances their risks to modern threats.

While the impact of megafauna extinctions over carnivore and herbivore communities has received a great deal of attention, only a handful of studies have assessed the loss of large vertebrates over scavengers to date^1,71^. We found evidence that suggests vultures responded demographically to changes in mammal communities, but no support for predator-prey interactions driving the historical demographic trajectory of obligate scavengers. These findings do not diminish the importance of carrion resources from mammalian predatory interactions^39^, but stress the behavioral plasticity of large vultures responding to ecological changes and the overestimated effect of food availability as a natural-limiting factor of some vulture populations. A consequence of the late Quaternary extinctions is that many extant species present large dietary breadths, even within specialized guilds^1^. Our findings suggest that, until recently, large avian scavengers survived because of this flexibility. This plastic foraging behavior, though, now exposes them to a suite of threats associated with current carrion resources^72^.

## Methods

To understand how changes in community composition impacted obligate scavengers, we evaluated the historical demography (100 KYA to mid-Holocene) of a tightly linked scavenger-predator-prey community module of southern South America. Additionally, we explored and compared how historical community changes affected the demographic trajectory of three large obligate scavengers inhabiting different continents that share life history strategies with long-generation times, and lifestyles involving social roosting and feeding habits, large individual home ranges and dependence on soaring flight^70^.

### Laboratory analysis

We extracted DNA from molted feathers of individual Andean Condors collected in 2013 from active roosting sites in northwestern Argentina: San Guillermo National Park (*n* = 11; −29.07°S, −69.35°W), La Payunia Provincial Reserve (*n* = 6; −36.40°S, −69.23°W) and Auca Mahuida Nature Reserve  (*n* = 6; −38°S, −68.70°W)^34,44^. We amplified the mitochondrial (mtDNA) complete Glu and partial control region with primers L16652-H621 (hereafter CR1), control region with L798-H1455 (hereafter CR2), and complete Phe and partial 12S with L798-H1795 (hereafter 12S)^45^. Additionally, we amplified exon 3 of the nuclear gene c-myc with the primers mycEX3D-RmycEX3D and mycEX3A-RmycEX3A^73^. Details on laboratory analyses are presented in the Supplementary material.

### Compiled datasets

We compiled mtDNA sequences from vicuñas^32^, guanacos^31^, pumas^30,47^, California condors^58^, white-backed vultures^74^ and outgroup species (*Puma yaguaroundi* and *Gyps ruepelli*) from GenBank (Supplementary Table S2). For demographic reconstruction of South American species, we selected samples that overlapped the geographic region with our Andean condor sampling sites based on haplotype structure from previous studies. In particular, puma samples came from southwestern South America, vicuña samples from Argentina and their southern Chilean range, and for guanacos we only considered the subspecies *L*. *g*. *guanicoe*. Samples of California condors were collected across their historical range^58^ and samples of white-backed vultures were collected in Africa, primarily Namibia^74^ (Supplementary Table S2). We tested for population panmixia by conducting exact test of population differentiation in Arlequin v3.5^75^ with 100,000 Markov chain, and eliminated samples of significantly segregated populations.

### Data analysis

We obtained Andean condor haplotype statistics via DNAsp v6.10.1^76^. For all following analysis, we used the Akaike information criterion corrected for sample size (AICc) to find the best fit evolutionary model with jModeltest 2.1.4^77^. Skylines plots and analyses for estimation of molecular clock rates were implemented in BEAST v.2.4.7^78^. Convergence to the stationary distribution and sufficient effective sampling sizes (>200) for each estimated parameter were checked using Tracer v1.5^79^, and four independent runs were combined using Log Combiner v2.4.7, a software implemented in BEAST2.

#### Substitution rates

We estimated clock rates for Andean and California condors implementing a Bayesian multispecies coalescent tree in *BEAST2^80^ using available mtDNA control region sequences from historical samples of California condors^58^ and Andean condor sequence generated in this study via CR1. The resulting molecular substitution rate for the Andean condor was used to estimate molecular evolution rates for CR2, 12S and c-myc via a coalescent constant population model process implemented in BEAST2. To estimate site and species-specific substitution rates for pumas, we also constructed multispecies coalescent analyses for loci ATP8 and NADH5 with *Puma yaguaroundi* as an outgroup^47^. We conducted a similar analysis to estimate cytochrome b oxidase I (cyt-b) substitution rate of *Gyps africanus* using *Gyps ruepelli* as an outgroup. For vicuñas and guanacos, mtDNA sequences from fossil samples and associated dates estimated by Metcalf *et al*.^10^ were used for calibration of fossilized birth-death models^81^ implemented in BEAST2 using the Sampled Ancestors add-on package^82^. For all analyses we compared the performance of a strict and uncorrelated relaxed lognormal clock model; we subsequently combined results of two independent runs of the best model (see details on the analysis in the Supplementary material).

#### Demographic analysis

We used three complimentary methods to infer changes in population size over time in our study species. We conducted neutrality tests, against null hypothesis of a constant population size, using Tajima’s *D*^83^, Fu’s *Fs*^84^, Fu and Li D* statistics in DNAsp v6.10.1^76^ with 10,000 coalescent simulations to calculate significance values. Second, we tested deviations from null models of constant populations via the distribution of pairwise sequence differences, or mismatch distribution, using the same software as above; observed versus expected results were plotted in R v3.4.2 (R Development Core Team 2017). For pumas and Andean condors, these two methods were implemented for concatenated mtDNA sequences. Finally, we estimated the timing and degree of population changes with Extended Bayesian Skyline Plot coalescent models (EBSP)^42^. These analyses depend on the estimation of evolutionary rates, which rely heavily on the statistical methods used to calibrate the clock^52^. To account for some of the uncertainty around the rates estimated, EBSP analyses were run using estimated molecular clock rates via log normal priors informed by median and 95% High Posterior Density (HPD) values from initial analyses (see Table 1 for further model details). We computed the posterior distribution of the number of demographic changes between runs, and formally rejected the null hypothesis of a constant population size when the 95% HPD of population changes excluded zero^42^. For species with conflicting results between EBSPs and neutrality test (Andean condors and white-backed vultures, see results), we obtained an approximated time since expansion based on mismatch analysis using the formula t = τ/2 µ, where µ = mµ is the mutation rate (as described previously) of the entire segment of m base pairs and τ is estimated based on the crest of the mismatch distribution^85,86^. Because we did not have a mutation rate for our entire mtDNA sequence of Andean Condors, we calculated the mean rate of the 3 fragments analyzed (µ_aver_ = 0.02), which was equal to the widely used substitution rate for mtDNA of birds^87^.

## Supplementary information

Supplementary material

## Data Availability

Details of laboratory and data analyses, and accession numbers for sequences retrieved from GenBank  have been uploaded as part of the Supplementary material. Additionally, Andean condor sequences generated in this study were archived in GenBank under the accession numbers MN031892-MN031983.
