# Supplementary material for: Demography of avian scavengers after Pleistocene megafaunal extinction
Source: Sci Rep. 2019 Jul 4;9:9680. doi: 10.1038/s41598-019-45769-w (PMC6609603; doi:10.1038/s41598-019-45769-w)
Supplement: Supplementary file 1 — Supplementary material [file 41598_2019_45769_MOESM1_ESM.pdf]

Demography of avian scavengers after Pleistocene megafaunal extinction

Paula L. Perrig[1*], Emily D. Fountain[1], Sergio A. Lambertucci[2] and Jonathan N. Pauli[1]

(1) Department of Forest and Wildlife Ecology, University of Wisconsin-Madison, 1630 Linden Dr., Madison, Wisconsin 53706, USA.

(2) Grupo de Investigaciones en Biología de la Conservación, INIBIOMA (Universidad de Comahue - CONICET), Quintral 1250, Bariloche, Rio Negro 8400, Argentina.

* Corresponding author: perrig@wisc.edu. ORCID 0000-0002-4269-9468

Sequencing of Andean condor samples

We extracted DNA from 23 Andean condor samples collected non-invasively by removing the blood clot at the superior umbilicus of each feather shaft [1]. We performed genomic isolation with QIAamp DNA Micro Kit (QIAGEN, Valencia, CA, USA) in a pre-PCR cleanroom facility at the University of Wisconsin dedicated to low template DNA, and included negative controls during the extractions. We amplified mitochondrial DNA (mtDNA), including the complete Glu and partial control region with primers L16652-H621 (CR1), control region with L798-H1455 (CR2), and complete Phe and partial 12S with L798-H1795 (12S) [2]. All PCRs were performed in a final volume of 20µl containing 2 µL template DNA, 1X PCR buffer, 0.25 mM dNTPs, 0.3µM of forward and reverse primer, and 0.025U taq polymerase (201203, Qiagen), and additional 0.5 µg/µl BSA for CR1 and CR2. For 12S, the PCR parameters consisted of 3 m denaturation at 94°C, 35 cycles of 30 s at 94°C, 30 s at 52°C and 30 s at 72°C, and a final extension step of 10 m at 72°C. For CR2 and CR1 the thermal profile included the same steps with an annealing temperature of 50°C and 54°C, respectively. Additionally, exon 3 of the nuclear gene c-myc was amplified with the primers mycEX3D-RmycEX3D and mycEX3A-RmycEX3A [3]. PCR products were purified using ExoSAPII (78201, Affymetrix) purification kit according to the manufacturer's instructions. We pair-end sequenced the fragments on an ABI 3730xl DNA analyzer at the Biotechnology Center, University of Wisconsin-Madison. Sequences were visually aligned using MEGA v7.0.26 [4] except for CR2 which was automatically aligned via webprank [5] under the default settings due to the presence of variable repeats.

Estimation of substitution rates

To estimate species-specific clock rates for condors, we combined available mtDNA control region sequences from historical samples of California condors (collected between 1825-1980; table S2) with our Andean condor sequences in a Bayesian multispecies coalescent analysis implemented in *BEAST2 [6]. We trimmed the data to have the same base pair length (sequence length 524 bp; California condors, n = 65, Andean condor, n = 23). The best fitting substitution model for the dataset was HKY+G (gamma shape 0.418) as estimated by JModeltest 2.1.4 [7]. A calibrated Yule tree prior was implemented with root height constrained at the estimated time of divergence of condors clade following Johnson et al. [8] by using a normal distribution with mean = 9.5 and std dev =1.6, and groups enforced as monophyletic. The MCMC was run for 90 million generations, sampling every 3000th generation. An improper Jeffrey's prior (1/X) was placed on the clock to allow calibration date to inform the clock rate. For this and all subsequent analysis, evaluation of model performances (convergence to the stationary distribution and effective sampling sizes >200) and resulting substitution rates were obtained using Tracer v1.5 [9]. Samples from two independent runs with a relaxed log normal clock were pooled and, after discarding the initial 20% as burn-in, a maximum clade credibility tree was compiled in TreeAnnotator v2.4.7 (included in the BEAST package) summarizing mean node heights. The tree was analyzed via FigTree to obtain median rates of sequence evolution and associated 95% HPD intervals for Andean and California condors independently.

The resulting molecular substitution rate for the Andean condor was used to estimate molecular evolution rates for CR2, 12S and nuclear gene c-myc using a coalescent constant population model process implemented in BEAST2. The model was run linking gene trees for mitochondrial loci and unlinked for nuclear locus, unlinked substitution and site models, and fix

substitution rate of 0.0129 for CR1 (as informed by previous analysis). The clock rate for the remaining loci was estimated from the CR1 clock using uniform clock rate priors (0 - ∞). Evolutionary models were HKY+I (prop. Inv. = 0.8680) for CR1, TN93 for CR2, and HKY for 12S and c-myc. The root height was constrained as described above. The MCMC was run for 90 million generations, sampling every 2500th generation. We pooled two independent runs of the model under a strict clock to obtain median and associated 95% HPD substitutions rates.

Two multispecies coalescent models were implemented to estimate substitution rates for pumas using the genes NADH5 [10,11] and ATP8 [10] from individuals across the Americas (n = 287) with *Puma yaguaroundi* as an outgroup [10]. We trimmed both datasets to have the same base pair length. To inform the tree, we grouped the haplotypes as North America, Central America and South America based on a neighbor joining tree created in Mega7 [4] and haplotype structure from previously published studies [10–12]. Both processes were run using an analytical population size model and a calibrated yule prior, with TRN evolutionary model for NADH and HKY model for ATP8, using a Jeffrey's prior (1/X) for clock rate estimates, a normal prior on the Most Recent Common Ancestor of the tree with mean 4.17 and standard deviation of 1 following Johnson et al. [13], and groups enforced as monophyletic [10]. Both MCMC were run for 90 million generations, sampling every 2500th generation. Models were tested under a strict and relaxed lognormal clock. Due to the standard deviation on the relaxed clock rate having a mean close to zero, as assessed in Tracer, we used a strict molecular clock. Two independent runs were pooled to obtain final substitution rates.

We estimated a substitution rate for cytochrome b (cytb) for *Gyps africanus* (n = 77) implementing a multispecies coalescent analysis in *BEAST2 along with sequences of *Gyps ruepelli* (n = 6, 1026 bp). The process was run under an analytical size integration population

model, calibrated yule tree prior with a birth rate of 3.67, Jeffrey's prior (1/X) clock rate, normal prior on the Most Recent Common Ancestor with mean 1 and standard deviation of 0.8, and with groups enforced as monophyletic [14]. The MCMC was run for 100 million generations, sampling every 3000th generation. Model performance was compared under a strict and relaxed log normal clock, and samples from two independent runs under a strict clock were pooled.

For both vicuñas and guanacos, mitochondrial DNA sequences from contemporary fossil samples (n = 3 and n = 25 samples of vicuñas and guanacos, respectively) and associated dates estimated by Metcalf et al. [15] were used for calibration of fossilized birth-death models [16] implemented in BEAST2 using the Sampled Ancestors add-on package [17]. Since the guanaco subspecies were paraphyletic [18], we only used data for *Lama guanicoe guanicoe* for estimating substitution rates (n = 265, 443 bp). For vicuñas, we did not find evidence of subspecies' differentiation, so we modelled all available data (n = 72, 458bp). Both analysis were parametrized using a origin date of species of 1.5 MYA according to [19]; thus, origin FBD was modeled in real space with mean 1.04, standard deviation 0.19, and offset 0.01062 for guanacos and 0.0215 for vicuñas. Sampling proportion prior had a beta distribution with both parameters set at 2 while diversification rate had an exponential prior with mean 1. We performed each analysis using 80 x 10^7 MCMC generations, sampling every 2500 generations, and two independent runs with a strict clock rate were combined to obtain a final rates for each camelid species.

Table S1. Substitution sites (S), number of haplotypes (h), haplotype diversity (hd), Tajima's *D* (D_T) and Fu's *Fs* (Fs) value and significance (bolded numbers) from coalescent simulations for 3 mitochondrial loci and one nuclear gene sequenced from 23 Andean condor samples collected in central Argentina.

	S	h	hd	D_T	p-value	Fs	p-value
c-myc	1	2	0.08	-1.16	0.270	-0.99	0.349
CR1	21	14	0.88	-1.82	**0.016**	-7.50	**<0.01**
CR2	12	2	0.08	-2.35	**<0.01**	3.21	0.905
12S	4	3	0.17	-1.88	**<0.01**	-0.78	0.378

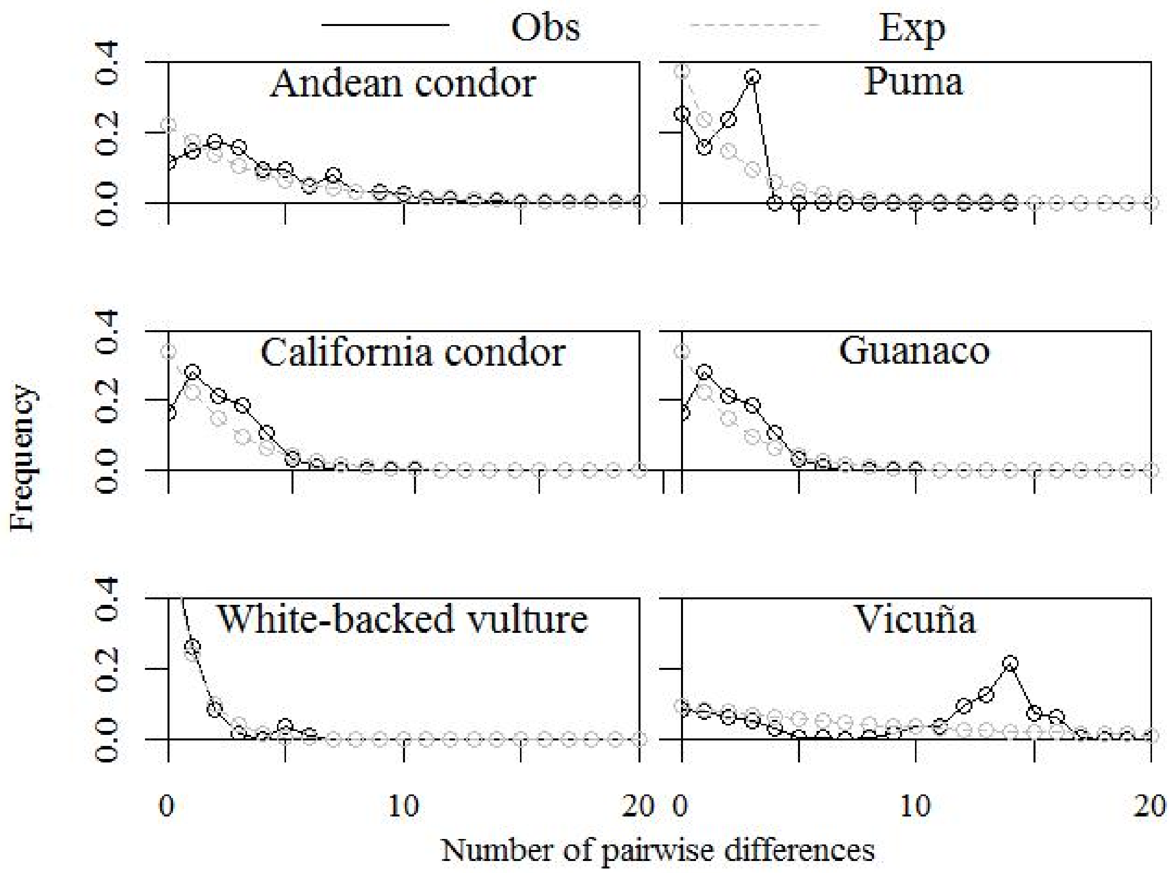

Figure S1. Observed and expected mismatch distribution for mitochondrial genes of all study species.

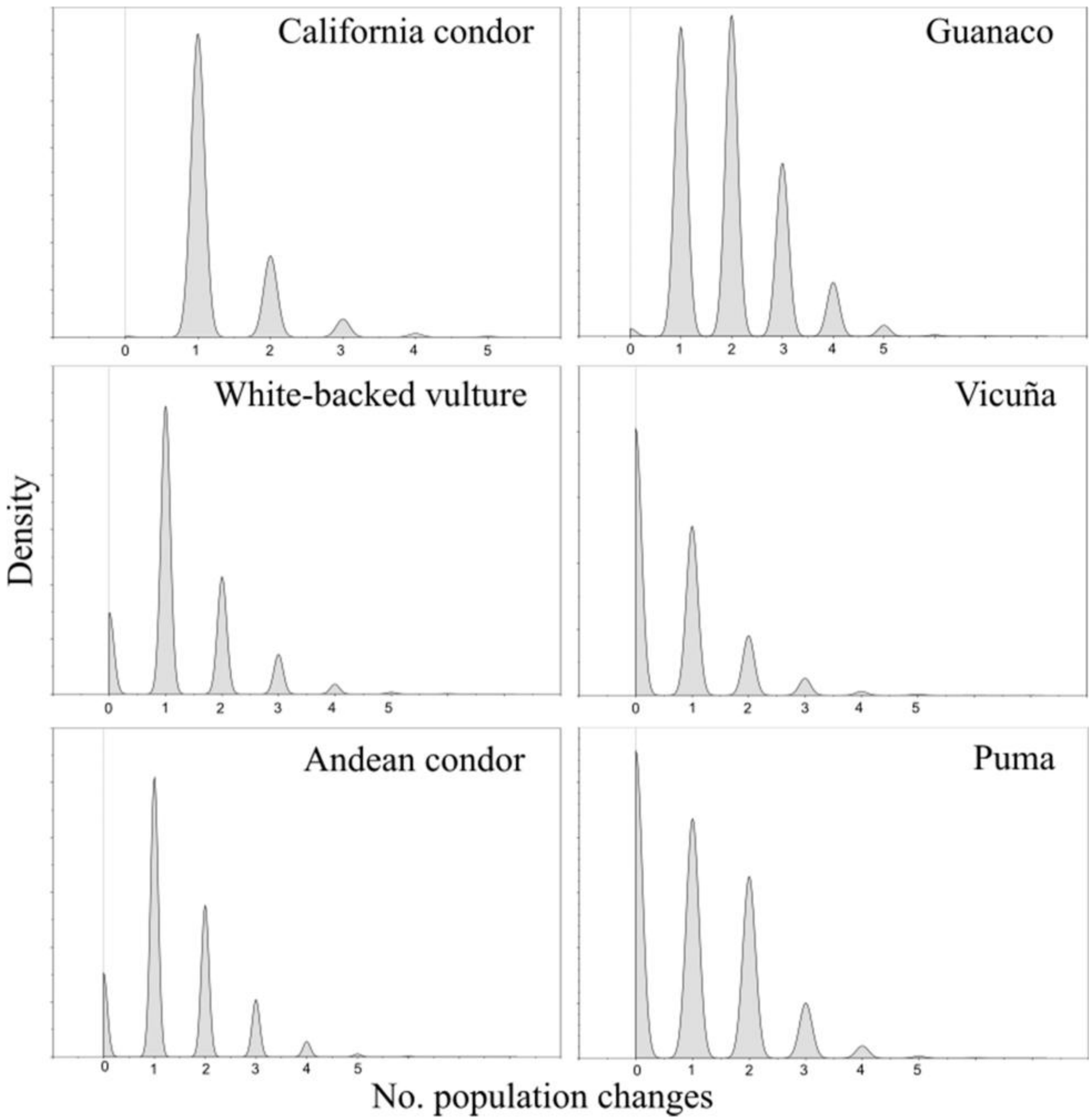

Figure S2. Number of population changes in the posterior distribution of Extended Bayesian Skyline Plots. A constant size coalescent model can be rejected when 0 population changes is outside the 95% Highest Posterior Density interval (HPD) [20]. This was the case for California condors (median of population changes = 1, 95% HPD = 1-3) and guanacos (2, 1-4), whereas we cannot rejected the null hypothesis of a constant population for white-backed vultures (1, 0-3), vicuñas (0, 0-2), Andean condors (1, 0-3) and pumas (1, 0-3).

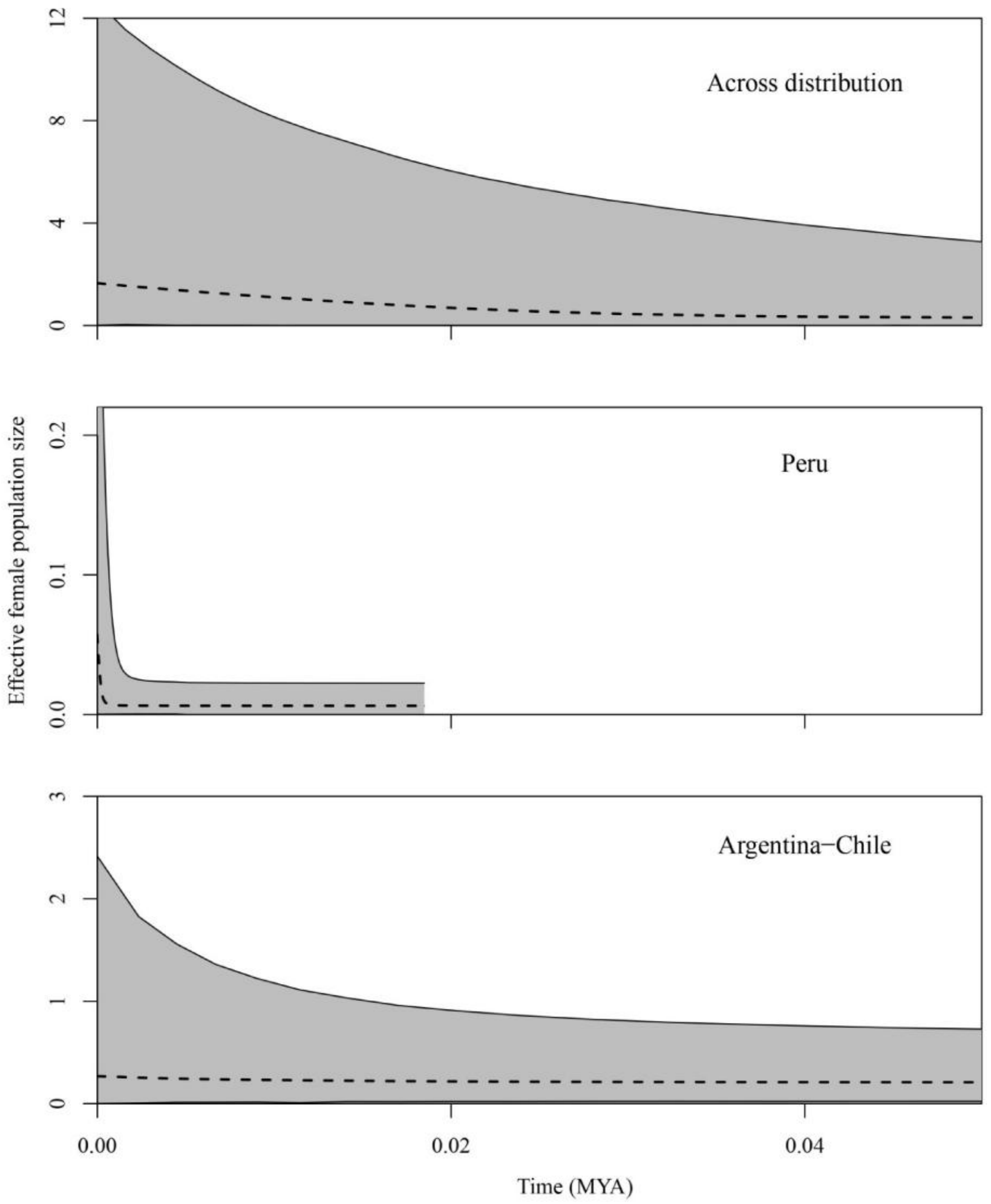

Figure S3. Extended Bayesian Skyline plots for vicuñas implemented with readily available D-loop sequences from (a) the complete species distribution (n = 72, 458 bp), (b) Perú (n = 206, 328 bp), and (c) southern range of vicuña distribution encompassing Chile and Argentina. The

models were parametrized as described in main text, except for the best evolutionary model that was HKY and 0.79 proportion invariant for Peruvian populations, and HKY with 0.024 gamma shape for the complete vicuña dataset. It is worth mentioning that pooling sequences from across the species distribution (a) can yield misleading results [21].

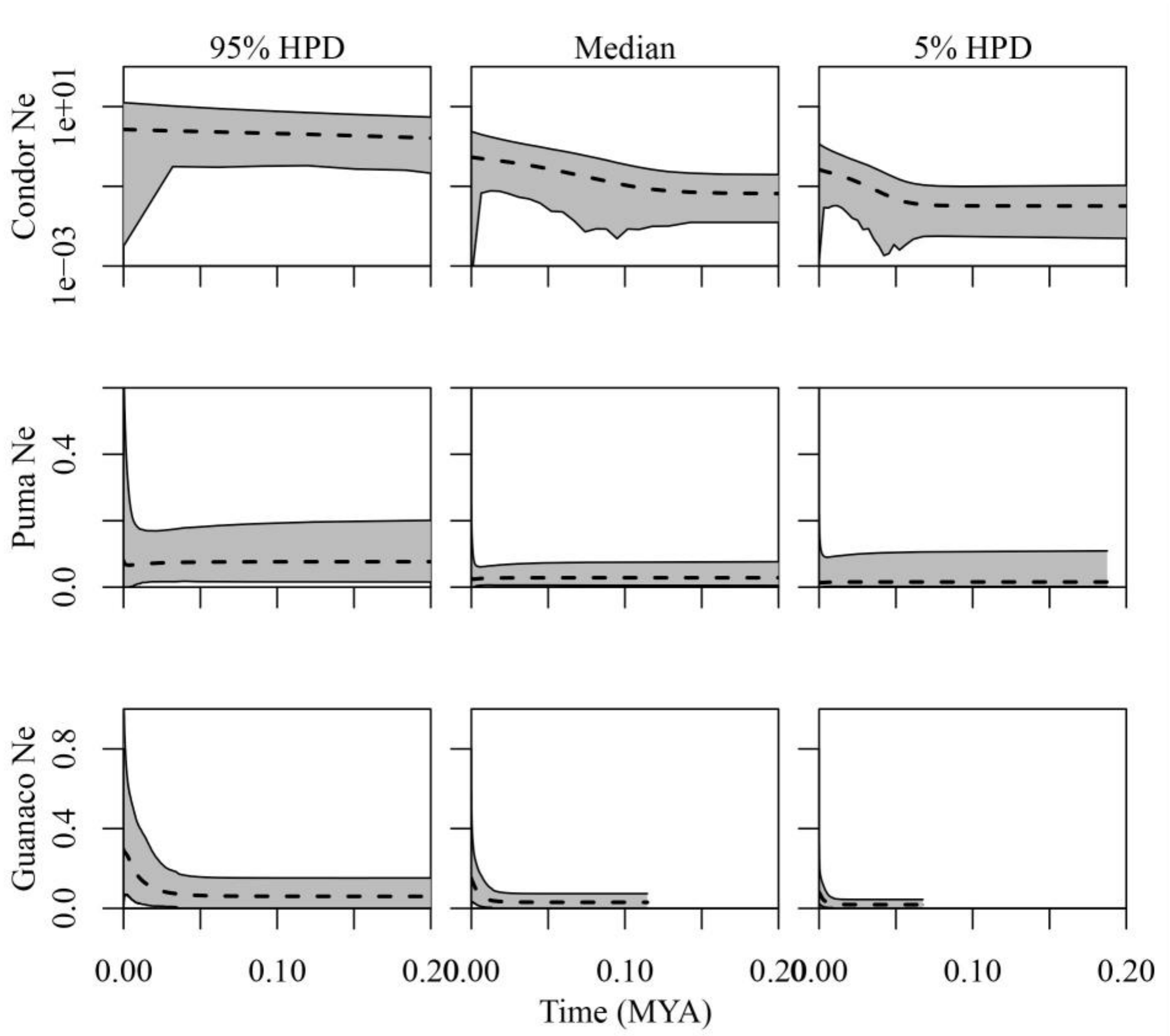

Figure S4. Evaluation of substitution rate effects on Extended Bayesian Skyline Plot (EBSP) analyses of Andean condors, pumas and guanacos' mitochondrial codons. The y-axis represents the female effective population size (Ne) multiplied by generation time, in log scale for Andean condors. EBSP analyses were conducted as described in main text but fixing molecular evolution rates at the lower (5% HPD), median and upper (95% HPD) values estimated in this study. Each plot shows the result of 4 independent runs combined after accounting for a 20% burn-in.

Table S2. Details of sequences retrieved from GenBank

Species	Haplotype	Origin	Locus	GenBank Accession #	# Inds	Reference
Vicugna vicugna		Argentina	CR	AY856304- AY856309, AY856319- AY856323	11	Marin et al. [22]
		Chile	CR	AY856310- AY856318, AY856339- AY856324	17	
		Peru	CR	AY856303- AY856270	33	
		Peru	D-loop	EF197725- EF197535	190	
		Chile	D-loop	EF197534- EF197502	30	
		Peru, Argentina	CR	KU753702- KU753673	4	Metcalf et al. [15]
Lama guanicoe		Argentina	CR	JX678410- JX678536, JX678400- JX678409	136	Marin et al. [18]
		Chile	CR	JX678537- JX678399, JX678301- JX678351	188	
		Bolivia	CR	JX678321- JX678340	19	
		Peru	CR	JX678291- JX678300	10	

		Argentina	CR	KU753671, KU753676- KU753679, KU753711- KU753715, KU753717, KU753718, KU753720-KU753722, KU753724, KU753725,	17	Metcalf et al. [15]
		Chile	CR	KU753701, KU753700, KU753707, KU753660	4	
		Peru	CR	KU753705, KU753706, KU753709, KU753710	4	
Puma concolor	Haplotype 18	South America	NADH5	KF460513	11	Matte et al. [11]
	Haplotype 19	South America	NADH5	KF460514	7	
	Haplotype 07	South America	NADH5	KF460502	5	
	Haplotypes A, L, F	South America	NADH5	AF241860	49	Culver et al. [10]
	Haplotype B	South America	NADH5	AF241861	1	

	Haplotype C	Central America	NADH5	AF241862	2	
	Haplotype D	South America	NADH5	AF241863	4	
	Haplotype E	South America	NADH5	AF241864	2	
	Haplotypes G, H	South America	NADH5	AF241865	10	
	Haplotypes I, J	South America	NADH5	AF241866	23	
	Haplotype K	South America	NADH5	AF241867	1	
	Haplotype M	North America	NADH5	AF241868	190	
	Haplotype N	North America	NADH5	AF241869	4	
	Haplotype A	South America	ATP8	AF241854	1	
	Haplotype B, D, E, F, G, K, L	South America	ATP8	AF241855	57	
	Haplotype C	Central America	ATP8	AF241856	2	
	Haplotypes H, I	South America	ATP8	AF241857	13	
	Haplotype J	South America	ATP8	AF241858	19	
	Haplotypes M, N	North America	ATP8	AF241859	194	
Puma yaguaroundi	JHA	Indet	Genome	KP202279	1	Li et al. Unpubl.

Gyps ruepelli	Haplotype GR52	France	Cytb	EU496452	1	Arshad et al. [23]
	Haplotype GR51	South Africa	Cytb	EU496451	1	
	Haplotype GR50	France	Cytb	EU496450	2	
	Haplotype GR53	Gambia	Cytb	DQ908973	1	Johnson et al. [14]
	Haplotype GR54	Gambia	Cytb	AY987260	1	Lerner & Mindell [24]
Gyps africanus	Haplotype GA1	Indet	Cytb	DQ908972	1	Johnson et al. [14]
	Haplotype GA2	Indet	Cytb	AY987263	1	Lerner & Mindell [24]
	Haplotype GA14	South Africa	Cytb	EU496414	1	Arshad et al. [23]
	Haplotype GA1	Namibia	Cytb	EU496401	31	
	Haplotype GA2	Namibia	Cytb	EU496402	18	
	Haplotype GA3	Namibia	Cytb	EU496403	10	
	Haplotype GA4	Namibia	Cytb	EU496404	3	
	Haplotype GA5	Namibia	Cytb	EU496405	2	
	Haplotype GA6	Namibia	Cytb	EU496406	2	
	Haplotype GA7	Namibia	Cytb	EU496407	2	
	Haplotype GA8	Namibia	Cytb	EU496408	1	

	Haplotype GA9	Namibia	Cytb	EU496409	1	
	Haplotype GA10	Namibia	Cytb	EU496410	1	
	Haplotype GA11	Namibia	Cytb	EU496411	1	
	Haplotype GA12	Namibia	Cytb	EU496412	1	
	Haplotype GA13	Namibia	Cytb	EU496413	1	
Gymnogyps californianus	Haplotype H18	USA	D-loop	KX379736	1	D'Elia et al. [25]
	Haplotype H17	USA	D-loop	KX379735	1	
	Haplotype H16	USA	D-loop	KX379734	1	
	Haplotype H15	USA	D-loop	KX379733	1	
	Haplotype H14	USA	D-loop	KX379732	1	
	Haplotype H13	USA	D-loop	KX379731	1	
	Haplotype H12	USA	D-loop	KX379730	1	
	Haplotype H11	USA	D-loop	X379729	1	
	Haplotype H10	USA	D-loop	KX379728	1	
	Haplotype H9	USA	D-loop	KX379727	1	
	Haplotype H8	USA	D-loop	KX379726	3	

Haplotype H7	USA	D-loop	KX379725	9
Haplotype H6	USA	D-loop	KX379724	8
Haplotype H5	USA	D-loop	KX379723	3
Haplotype H4	USA	D-loop	KX379722	13
Haplotype H3	USA	D-loop	KX379721	1
Haplotype H2	USA	D-loop	KX379720	1
Haplotype H1	USA	D-loop	KX379719	20

References

1. Horváth, M., Martínez-Cruz, B., Negro, J. J., Kalmar, L. & Godoy, J. A. An overlooked DNA source for non-invasive genetic analysis in birds. *J. Avian Biol.* **36**, 84–88 (2005).
2. Hendrickson, S. *et al.* Low Genetic Variability in the Geographically Widespread Andean Condor. *Condor* **105**, 1–12 (2003).
3. Ericson, P. G. P. *et al.* Diversification of Neoaves: integration of molecular sequence data and fossils. *Biol. Lett.* **2**, 543–7 (2006).
4. Kumar, S., Stecher, G. & Tamura, K. MEGA7: Molecular Evolutionary Genetics Analysis version 7.0 for bigger datasets. *Mol. Biol. Evol.* **33**, 1870–1874 (2016).
5. Löytynoja, A. & Goldman, N. webPRANK: a phylogeny-aware multiple sequence aligner with interactive alignment browser. *BMC Bioinformatics* **11**, 579 (2010).
6. Ogilvie, H. A., Bouckaert, R. R. & Drummond, A. J. StarBEAST2 Brings faster species tree inference and accurate estimates of substitution rates. *Mol. Biol. Evol.* **34**, 2101–2114 (2017).
7. Darriba, D., Taboada, G. L., Doallo, R. & Posada, D. jModelTest 2: more models, new heuristics and parallel computing. *Nat. Methods* **9**, 772–772 (2012).
8. Johnson, J. A., Brown, J. W., Fuchs, J. & Mindell, D. P. Multi-locus phylogenetic inference among New World Vultures (Aves: Cathartidae). *Mol. Phylogenet. Evol.* **105**, 193–199 (2016).
9. Rambaut, A. & Drummond, A. Tracer v1.4. http://tree.bio.ed.ac.uk/software/tracer/ (2007).
10. Culver, M., Johnson, W. E., Pecon-slattery, J. & O’Brien, S. J. Genomic ancestry of the American puma (*Puma concolor*). *J. Hered.* **91**, 186–197 (2000).

11. Matte, E. M. *et al.* Molecular evidence for a recent demographic expansion in the puma (*Puma concolor*) (Mammalia, Felidae). *Genet. Mol. Biol.* **36**, 586–597 (2013).

12. Caragiulo, A., Dias-Freedman, I., Clark, J. A., Rabinowitz, S. & Amato, G. Mitochondrial DNA sequence variation and phylogeography of Neotropic pumas (Puma concolor). *Mitochondrial DNA* **1736**, 1–9 (2013).

13. Johnson, W. E. *et al.* The late Miocene radiation of modern felidae: A genetic assessment. *Science* **311**, 73–77 (2006).

14. Johnson, J. A., Lerner, H. R. . L., Rasmussen, P. C. & Mindell, D. P. Systematics within *Gyps* vultures: a clade at risk. *Bmc Evol. Biol.* **6**, 65 (2006).

15. Metcalf, J. L. *et al.* Synergistic roles of climate warming and human occupation in Patagonian megafaunal extinctions during the Last Deglaciation. *Sci. Adv.* **2**, 1–9 (2016).

16. Heath, T. A, Huelsenbeck, J. P. & Stadler, T. The fossilized birth–death process for coherent calibration of divergence-time estimates. *Proc. Natl. Acad. Sci.* **111**, E2957–E2966 (2014).

17. Gavryushkina, A., Welch, D., Stadler, T. & Drummond, A. J. Bayesian Inference of Sampled Ancestor Trees for Epidemiology and Fossil Calibration. *PLoS Comput. Biol.* **10**, e1003919 (2014).

18. Marin, J. C., González, B. A., Poulin, E., Casey, C. S. & Johnson, W. E. The influence of the arid Andean high plateau on the phylogeography and population genetics of guanaco (*Lama guanicoe*) in South America. *Mol. Ecol.* **22**, 463–482 (2013).

19. Lameiro, A. M. D. Evolutionary Origins and Domestication of South American camelids, the alpaca (*Vicugna pacos*) and the llama (*Lama glama*) explained through molecular DNA methods. PhD Dissertation, State University of New York (2016).

20. Heled, J. & Drummond, A. J. Bayesian inference of population size history from multiple loci. *BMC Evol. Biol.* **8**, 289 (2008).

21. Grant, W. S. Problems and cautions with sequence mismatch analysis and Bayesian skyline plots to infer historical demography. *J. Hered.* **106**, 333–346 (2015).

22. Marín, J. C. *et al.* Mitochondrial phylogeography and demographic history of the Vicuña: implications for conservation. *Heredity* **99**, 70–80 (2007).

23. Arshad, M. *et al.* Genetic variation of four *Gyps* species (*Gyps bengalensis*, *G. africanus*, *G. indicus* and *G. fulvus*) based on microsatellite analysis. *J. Raptor Res.* **43**, 227–236 (2009).

24. Lerner, H. R. L. & Mindell, D. P. Phylogeny of eagles, Old World vultures, and other Accipitridae based on nuclear and mitochondrial DNA. *Mol. Phylogenet. Evol.* **37**, 327–346 (2005).

25. D'Elia, J., Haig, S. M., Mullins, T. D. & Miller, M. P. Ancient DNA reveals substantial genetic diversity in the California Condor (*Gymnogyps californianus*) prior to a population bottleneck. *Condor* **118**, 703–714 (2016).
